# FRET-based biosensor moxCRONOS enables quantitative monitoring of macromolecular crowding in organelles and protein aggregates

**DOI:** 10.1093/jb/mvaf056

**Published:** 2025-09-30

**Authors:** Yurina Nakajima, Hiroaki Suzuki, Tamami Miyagi, Kohsuke Kanekura

**Affiliations:** Department of Pharmacology, Tokyo Medical University, 6-1-1 Shinjuku, Shinjuku-ku, Tokyo 160-8402, Japan; Department of Pharmacology, Tokyo Medical University, 6-1-1 Shinjuku, Shinjuku-ku, Tokyo 160-8402, Japan; Department of Pharmacology, Tokyo Medical University, 6-1-1 Shinjuku, Shinjuku-ku, Tokyo 160-8402, Japan

**Keywords:** biosensor, FRET, LLPS, macromolecular crowding, protein aggregation

## Abstract

Macromolecular crowding is a fundamental property of the intracellular environment that influences protein folding, enzymatic activity, and phase behavior. Disruptions to the homeostasis of macromolecular crowding can drive pathological processes, such as aberrant liquid–liquid phase separation and protein aggregation, which are central features of several neurodegenerative diseases. However, tools for quantifying crowding and aggregation remain limited. Here, we describe moxCRONOS, a Förster resonance energy transfer (FRET)-based biosensor that enables the quantitative measurement of macromolecular crowding and protein condensation. moxCRONOS retains the optical properties of the original CRONOS sensor but offers enhanced stability in oxidative environments, such as within the endoplasmic reticulum or under sodium arsenite treatment, allowing for direct comparison of crowding levels across organelles regardless of redox conditions. Moreover, when fused to dipeptide repeat proteins associated with *C9ORF72*-linked neurodegeneration, moxCRONOS detects aggregation-prone states—especially in cells expressing glycine–alanine (GA) repeats. Using fluorescence-activated cell sorting, we achieved sensitive and quantitative detection of heterogeneous high-FRET cell populations containing GA aggregates. FRET signal intensity increased upon treatment with a molecular crowding agent or a proteasome inhibitor. These findings establish moxCRONOS as a versatile biosensor for investigating both physiological macromolecular crowding and pathological protein aggregation, with significant potential for disease modeling and therapeutic screening.

## Graphical Abstract

**Graphical Abstract:**
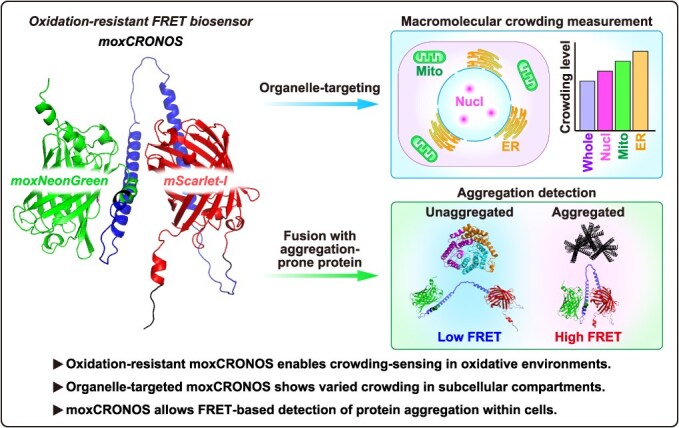

The intracellular environment is densely packed with macromolecules, creating macromolecular crowding *(**1**)*. This crowded state affects molecular diffusion, protein folding, enzymatic activity, and phase behavior *(**2**)*. Recently, macromolecular crowding has been linked to liquid–liquid phase separation (LLPS), which organizes membrane-less cellular compartments *(**3**)*. Aberrant LLPS and pathological protein aggregation are key features of neurodegenerative diseases, including amyotrophic lateral sclerosis and frontotemporal dementia *(**4**)*, where dense, often irreversible, aggregates disrupt proteostasis and drive disease.

Despite its importance, crowding levels within specific subcellular compartments remain poorly understood, partly due to limited tools for quantitative assessment. Similarly, methods for objectively quantifying protein aggregation or condensate formation remain underdeveloped, especially for subtle, dynamic, or rare events.

Förster resonance energy transfer (FRET)-based biosensors have become valuable tools for detecting physical changes in the intracellular environment *(**5**,*  *6**)*. The crowding-sensitive sensor CRONOS, which utilizes FRET between mNeonGreen and mScarlet-I, can detect shifts in macromolecular packing *(**7**)*. Here, we employed a modified version, moxCRONOS, in which a redox-sensitive cysteine was replaced with serine to avoid the possibility of disulfide bond formation as well as other oxidative modifications and to potentially improve stability in oxidative compartments, such as the endoplasmic reticulum (ER) *(**8**)*. Because this modification enhances sensor robustness, we employed moxCRONOS to monitor macromolecular crowding and condensate formation across distinct compartments and under disease-relevant aggregation conditions. We further combined moxCRONOS with flow cytometry (fluorescence-activated cell sorting, FACS) for high-throughput quantitative FRET analysis. Compared with microscopy, FACS offers objective, population-level measurements and can detect rare cell subpopulations exhibiting protein aggregation. This platform enables versatile investigation of physiological and pathological crowding states in cell biology and disease research.

## Materials and Methods

### Plasmid constructions

cDNAs encoding CRONOS and moxCRONOS (C149S) were synthesized by GenScript (Piscataway, NJ, USA) and subcloned into the *Escherichia coli* expression vector pET28a(+). For mammalian expression, CRONOS and moxCRONOS were used to replace EGFP in the pEGFP-C1 vector. Dipeptide repeat protein (DPR) fusion constructs were generated by replacing EGFP in EGFP-FLAG-DPRs *(**9**)* with moxCRONOS. moxCRONOS-NPM1 was constructed by fusing the full-length NPM1 to its C-terminus. For ER targeting, moxCRONOS was synthesized with an N-terminal prolactin signal (MDSKGSSQKGSRLLLLLVVSNLLLCQGVVSTGPVAT) and a C-terminal ER retention KDEL motif and subcloned into pcDNA3.1(+). To generate mitochondria-targeting moxCRONOS, the COX8-derived 4 × mts sequence was PCR amplified from p4 × mts-mScarlet3_N1, a gift from Dr. Dorus Gadella, University of Amsterdam (Addgene plasmid # 189774; RRID:Addgene_189774) *(**10**)*, and inserted at the N terminus of moxCRONOS. The pEGFP-C1-FLAG-GA_200_ vector was constructed by inserting the synthesized FLAG-GA_200_ sequence (GenScript) into the pEGFP-C1 vector. moxCRONOS-FLAG-GA_200_ was generated by replacing EGFP in pEGFP-C1-FLAG-GA_200_ with moxCRONOS. The vector for FLAG-VAPB was from a previous study *(**11**)*.

### Antibodies and compounds

FLAG antibody (Cat# F1804, RRID:AB_262044) was purchased from Sigma-Aldrich (St. Louis, MO, USA). mNeonGreen antibody (Cat# 29523-1-AP, RRID:AB_3086140) and Tom20 antibody (Cat# 11802-1-AP; RRID:AB_2207530) were obtained from Proteintech Group (Rosemont, IL, USA). X-box-binding protein 1 (XBP-1) antibody (Cat# sc-7160, RRID:AB_794171) was acquired from Santa Cruz Biotechnology (Dallas, TX, USA). Glyceraldehyde 3-phosphate dehydrogenase (GAPDH) antibody (Cat# 2118; RRID:AB_561053) was purchased from Cell Signaling Technology (Danvers, MA, USA). Polyethylene glycol (PEG)4000 was purchased from Tokyo Chemical Industry Co., Ltd (Tokyo, Japan). Ficoll was purchased from NACALAI TESQUE, INC. (Kyoto, Japan). Sodium arsenite (NaAsO₂) was purchased from FUJIFILM Wako Pure Chemical Corporation (Osaka, Japan). Thapsigargin, tunicamycin, and MG132 were acquired from Sigma-Aldrich.

### Cell culture and transfection

HeLa cells were purchased from ATCC (Manassas, VA, USA; CCL-2, RRID:CVCL_0030). The cells were grown in Dulbecco’s modified Eagle’s medium (FUJIFILM Wako Pure Chemical Corporation) supplemented with 10% fetal bovine serum (Thermo Fisher Scientific, Waltham, MA, USA). The absence of mycoplasma contamination was validated using a PCR Mycoplasma Detection Set (TaKaRa Bio, Shiga, Japan). Transfection was performed using Lipofectamine 2000 (Thermo Fisher Scientific) under the manufacturer’s protocol.

### Preparation of recombinant CRONOS proteins

pET28a(+)-CRONOS and pET28a(+)-moxCRONOS were transformed into *E. coli* BL21 cells and plated on LB agar containing kanamycin. After overnight incubation at 37°C, a single colony was cultured in LB-kanamycin at 37°C until mid-log phase, then protein expression was induced with 0.1 mM isopropyl β-d-1-thiogalactopyranoside and incubated overnight at 30°C with shaking. Bacterial pellets were resuspended in lysis buffer (50 mM Tris–HCl, pH 7.4, 500 mM NaCl, 1% Triton X-100, protease inhibitor) supplemented with lysozyme (FUJIFILM Wako Pure Chemical Corporation) and lysed by sonication on ice. The recombinant CRONOS proteins were purified with His60 Ni beads (TaKaRa Bio) following the manufacturer’s protocol. After elution by imidazole-containing buffer, the recombinant CRONOS proteins were dialyzed with PBS using a Slide-A-Lyzer MINI (Thermo Fisher Scientific). Fluorescence spectra were recorded on an RF-6000 spectrophotometer (Shimadzu, Kyoto, Japan) with 506 nm excitation of mNeonGreen or moxNeonGreen.

### Western blotting analysis

Cells were lysed in a 4% SDS-containing sample buffer by sonication. The samples in the SDS-containing sample buffer were boiled at 95°C for 5 min, fractionated by SDS-PAGE, and blotted onto poly-vinylidene fluoride membranes (Pall Corporation, Port Washington, NY, USA). After blocking with 5% skim milk (Becton, Dickinson and Company, Sparks, MD, USA) in Tris-buffered saline with 0.1% Tween 20, immunoblotting was performed with indicated antibodies. Immunoreactive bands were detected with ECL Western Blotting Detection Reagents (GE Healthcare UK Ltd, Buckinghamshire, England) using ChemiDoc Touch MP (Bio-Rad Laboratories, Hercules, CA, USA). GAPDH was used as an internal control.

### FACS analysis

Cells fixed with 20% formalin (FUJIFILM Wako Pure Chemical Corporation) were analyzed using FACS (FACSCanto II; BD Biosciences, Franklin Lakes, NJ, USA). Compensation was conducted using cells individually expressing either moxNeonGreen or mScarlet-I to correct for spectral overlap between the fluorescent proteins. Single-positive controls were used to generate the compensation matrix. All subsequent analyses were performed using these compensation settings. In the gating strategy, cell populations were initially identified and debris was excluded based on size (FSC-A) versus granularity (SSC-A) plots. Singlet cells were selected using FSC-H versus FSC-W and SSC-H versus SSC-W. Fluorescence-positive cells were identified by comparison with an empty vector-transfected negative control. Excitation was performed at 488 nm, and fluorescence was detected using the fluorescein isothiocyanate and phycoerythrin channels. Data were analyzed using FlowJo software (BD Biosciences).

### Confocal laser microscopy

HeLa cells were fixed in 20% formalin, permeabilized with 0.1% Triton X-100 in PBS, and blocked with 0.1% bovine serum albumin in PBS. For immunostaining, cells were incubated with FLAG or Tom20 antibodies, followed by Alexa Fluor 647-labeled goat anti-mouse IgG (Cat# A-21236, RRID: AB_2535805, Thermo Fisher Scientific) or anti-rabbit IgG (Cat# A-21245, RRID: AB_2535813, Thermo Fisher Scientific). Nuclei were counterstained with 4′,6-diamidino-2-phenylindole (DAPI; DOJINDO LABORATORIES, Kumamoto, Japan), and samples were mounted with ProLong Gold (Thermo Fisher Scientific). For samples without antibody staining, the cells were fixed, stained with DAPI, and mounted in a similar manner. All samples were observed and analyzed by confocal laser scanning microscopy using a model LSM710 instrument with ZEN 2010 software (Carl Zeiss, Oberkochen, Germany). For FRET analysis, cells were excited at 488 nm, and emission from moxNeonGreen was detected between 520 and 560 nm. Emissions from mScarlet-I were detected between 590 and 712 nm. The FRET efficiency was calculated using the mScarlet-I/moxNeonGreen emission ratio. Scale bars in all images represent 10 μm.

### FRAP analysis

FRAP analysis was performed using an LSM710 confocal laser scanning microscope equipped with a 63× water immersion objective. Cells were maintained at 37°C during imaging. A defined region of interest was photobleached using 100% laser power after acquiring two prebleach images. Fluorescence recovery was monitored at 2-s intervals for up to 1 min.

### Statistical analyses

All values in the figures are shown as mean ± SD. Statistical analyses were performed in triplicate using one-way analysis of variance, followed by a *post hoc* test (Tukey’s multiple comparison test) or two-tailed Student’s *t*-test. All statistical analyses were performed using Prism 8 software (ver. 8.4.3) (^*^P < 0.05; ^**^P < 0.01; ^***^P < 0.001; ^****^P < 0.0001).

## Results

### moxCRONOS preserves the macromolecular crowding-sensing capability

**Fig. 1:**
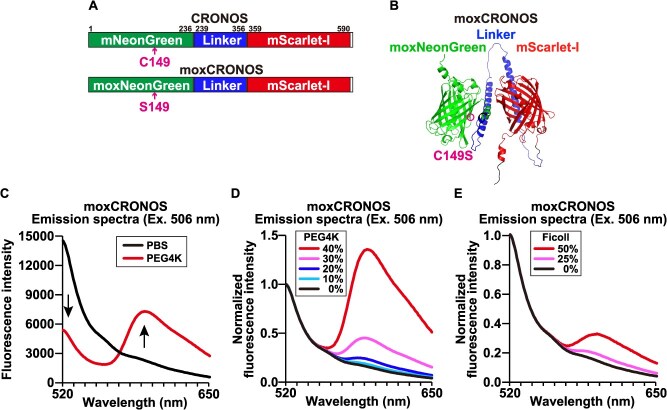
**moxCRONOS preserves the macromolecular crowding-sensing capability.** (A) Schematic representations of the CRONOS and moxCRONOS protein structures. CRONOS consists of mNeonGreen and mScarlet-I fused with a linker. In moxCRONOS, the cysteine residue at position 149 is substituted with serine (C149S). (B) Predicted folded structure of moxCRONOS generated by AlphaFold3. The structure shows moxNeonGreen (green) and mScarlet-I (red) assembled with the linker (blue) at the center. The C149S substitution is highlighted in magenta. (C) Fluorescence spectra of recombinant moxCRONOS dissolved in PBS (black line) or PBS containing 40% PEG4000 (4 K) (red line) upon excitation at 506 nm. (D) Fluorescence spectra of recombinant moxCRONOS dissolved in PBS or PBS containing 0–40% PEG4000 (4 K), upon excitation at 506 nm. Fluorescence intensity is normalized to the value at 520 nm. (E) Fluorescence spectra of recombinant moxCRONOS dissolved in PBS or PBS containing 0%, 25%, or 50% Ficoll, upon excitation at 506 nm. Fluorescence intensity is normalized to the value at 520 nm.

To enable the accurate measurement of macromolecular crowding by CRONOS regardless of the redox state, we developed a monomeric oxidizing environment-resistant variant, termed moxCRONOS. This engineered sensor retains proper folding and fluorescence even in oxidative environments, such as the ER or secretory pathways *(**8**)*. Within the original CRONOS sequence, the cysteine at position 149 (C149) is the sole cysteine residue and is susceptible to disulfide bond formation and other undesirable modifications. To prevent these modifications, C149 was replaced with a serine (S149) (Fig. 1A and B) *(**12**)*.

To determine whether moxCRONOS retained the fluorescence properties of the original CRONOS, we analyzed the fluorescence properties of recombinant moxCRONOS. As previously reported for CRONOS, moxCRONOS exhibits decreased fluorescence at approximately 520 nm and a corresponding increase near 590 nm upon excitation at 506 nm when treated with polyethylene glycol (PEG) 4000 to enhance macromolecular crowding (Fig. 1C) *(**7**)*. This spectral shift reflected the FRET from moxNeonGreen to mScarlet-I. The PEG-induced spectral shift was concentration dependent (Fig. 1D), and a similar concentration-dependent spectral shift was also observed when Ficoll was used as the crowding agent (Fig. 1E). These results indicated that, similar to the original CRONOS, moxCRONOS responds sensitively to macromolecular crowding in a concentration-dependent manner.

### moxCRONOS retains stable FRET efficiency under oxidative conditions

**Fig. 2:**
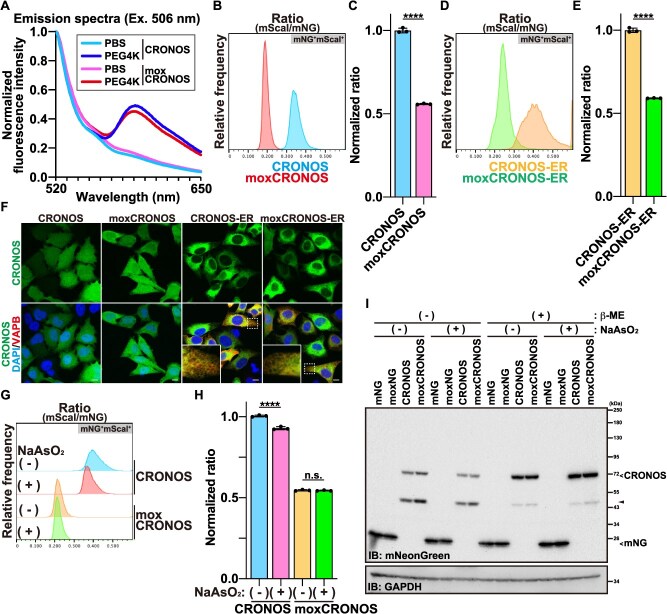
**moxCRONOS is more stable under oxidative conditions than CRONOS.** (A) Fluorescence spectra of recombinant CRONOS and moxCRONOS dissolved in PBS or PBS containing 30% PEG4000 (4 K), upon excitation at 506 nm. Fluorescence intensity is normalized to the value at 520 nm. (B, C) HeLa cells were transfected with either CRONOS or moxCRONOS. After 48 h, cells were fixed and analyzed by FACS. (B) Histograms showing the distribution of the mScarlet-I/mNeonGreen (mScaI/mNG) fluorescence intensity ratio within the mNG^+^mScaI^+^ population. (C) Median values of the mScarlet-I/mNeonGreen ratio calculated from the mNG^+^mScaI^+^ population shown in (B). (D, E) HeLa cells were transfected with either CRONOS-ER or moxCRONOS-ER. After 48 h, cells were fixed and analyzed by FACS. (D) Histograms showing the distribution of the mScarlet-I/mNeonGreen (mScaI/mNG) fluorescence intensity ratio within the mNG^+^mScaI^+^ population. (E) Median values of the mScarlet-I/mNeonGreen ratio calculated from the mNG^+^mScaI^+^ population shown in (D). (F) HeLa cells were transfected with CRONOS, moxCRONOS, CRONOS-ER, or moxCRONOS-ER. After 48 h, cells were fixed and observed using confocal laser scanning microscopy. For ER localization, cells were co-transfected with FLAG-tagged VAPB and immunostained with an anti-FLAG antibody. The image within the solid white line is an enlarged view of the area enclosed by the dotted line. (G, H) HeLa cells were transfected with either CRONOS or moxCRONOS. After 48 h, cells were treated with 500 μM NaAsO_2_ for 1.5 h, fixed, and analyzed by FACS. (G) Histograms showing the distribution of the mScarlet-I/mNeonGreen (mScaI/mNG) fluorescence intensity ratio within the mNG^+^mScaI^+^ population. (H) Median values of the mScarlet-I/mNeonGreen ratio calculated from the mNG^+^mScaI^+^ population shown in (G). n.s.: not significant. (I) HeLa cells were transfected with mNeonGreen (mNG), moxNeonGreen (moxNG), CRONOS, or moxCRONOS. After 48 h, cells were treated with 500 μM NaAsO_2_ for 1.5 h, harvested, and lysed in SDS-containing lysis buffer in the presence or absence of β-mercaptoethanol (β-ME). Following sonication, the cell lysates were subjected to SDS-PAGE and subsequently analyzed by immunoblotting (IB) with the indicated antibodies. Arrowhead indicates an uncharacterized protein, most likely a degradation product of CRONOS.

**Fig. 3:**
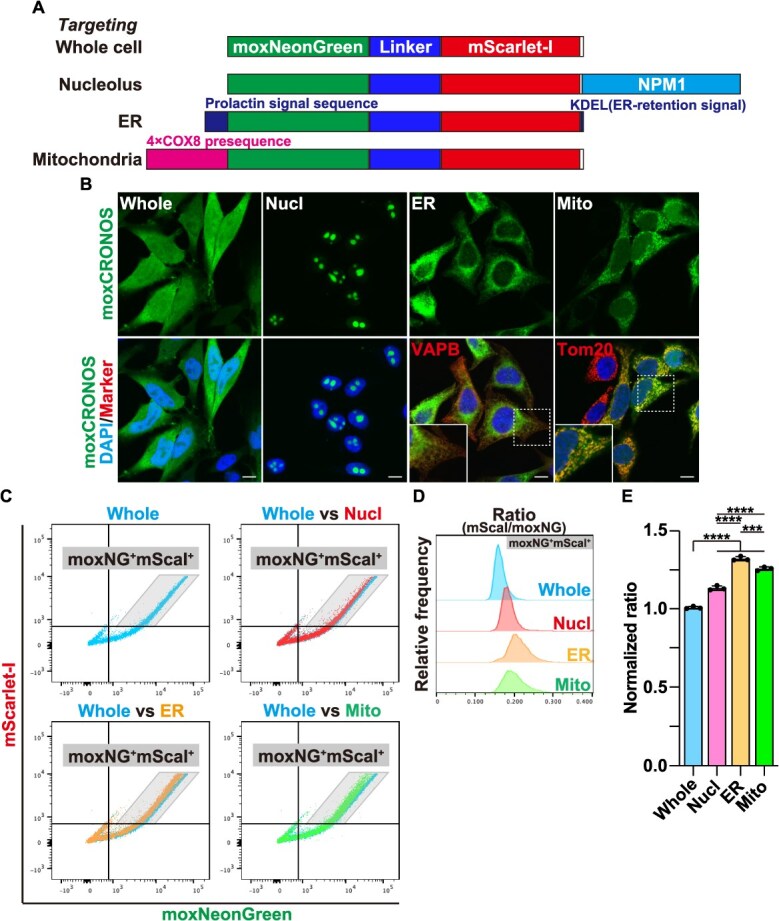
**Organelle-targeted moxCRONOS reveals compartment-specific differences in molecular crowding.** (A) Schematic representation of moxCRONOS protein constructs designed to localize to specific subcellular organelles. (B) HeLa cells were transfected with moxCRONOS variants targeted to different subcellular organelles (Nucl; nucleolus, Mito; mitochondria). After 48 h, cells were fixed and observed using confocal laser scanning microscopy. For ER localization, cells were co-transfected with FLAG-tagged VAPB and immunostained with an anti-FLAG antibody. Mitochondria were visualized by immunostaining with an anti-Tom20 antibody. The image within the solid white line is an enlarged view of the area enclosed by the dotted line. (C–E) HeLa cells were transfected with moxCRONOS variants targeting different subcellular organelles. After 48 h, cells were fixed and analyzed by FACS. (C) Dot plots show fluorescence intensities of moxNeonGreen (*x*-axis) and mScarlet-I (*y*-axis). (D) Histograms showing the distribution of the mScarlet-I/moxNeonGreen (mScaI/moxNG) fluorescence intensity ratio within the moxNG^+^mScaI^+^ population delineated by the gray parallelogram in (C). (E) Median values of the mScarlet-I/moxNeonGreen ratio calculated from the moxNG^+^mScaI^+^ population shown in (D).

We next examined whether the substitution of C149 with serine improves the performance of CRONOS in oxidizing environments. *In vitro* assays using PEG4000-induced crowding showed that both CRONOS and moxCRONOS responded similarly, although original CRONOS exhibited slightly higher FRET efficiency (Fig. 2A), suggesting that the mutation modestly reduces the baseline but does not impair crowding sensitivity. In mammalian cells, flow cytometric analysis using the mScarlet-I to mNeonGreen fluorescence intensity ratio upon excitation at 488 nm as an indicator of FRET revealed higher FRET efficiency for original CRONOS compared to moxCRONOS (Fig. 2B and C). The same trend was observed when the sensors were targeted to the ER (Figs 2D, E, and  3A). Confocal microscopy confirmed correct ER localization of the constructs (Fig. 2F). This reduction in FRET efficiency of moxCRONOS is likely attributable, at least in part, to the lower fluorescence quantum yield of moxNeonGreen compared to mNeonGreen, as previously reported *(**8**)*.

A clear difference in measurement robustness was observed under oxidative stress. Treatment with sodium arsenite significantly decreased FRET efficiency of original CRONOS, whereas moxCRONOS remained stable (Fig. 2G and H). Western blotting under non-reducing conditions showed no evidence of dimer formation as well as high molecular weight proteins under oxidative stress conditions (Fig. 2I), indicating that intermolecular disulfide bonds are unlikely to be involved. Since C149 is the only cysteine residue in CRONOS, these results suggest that oxidative modifications at this site—such as S-glutathionylation, S-sulfenylation, S-nitrosylation, or higher oxidation states (sulfinylation/sulfonylation)—may perturb the sensor conformation and reduce FRET independently of crowding *(**13**)*. By eliminating this reactive thiol, moxCRONOS avoids redox interference and maintains a specific response to macromolecular crowding even in oxidizing conditions.

### Organelle-targeted moxCRONOS enables detection of macromolecular crowding in intracellular compartments

Using moxCRONOS, we investigated macromolecular crowding in distinct subcellular compartments. Organelle-specific targeting signals were fused to moxCRONOS to direct them to various locations. moxCRONOS, which lacks a targeting sequence, shows diffuse localization throughout the cell. Nucleolar targeting was achieved by fusing the nucleolar protein nucleophosmin 1 (NPM1) to the C-terminus of moxCRONOS. To target ER, an N-terminal prolactin signal sequence and C-terminal KDEL ER retention signal were attached. Mitochondrial localization was accomplished by appending four repeats of the COX8 presequence to the N terminus of moxCRONOS (Fig. 3A). Confocal imaging confirmed the correct localization of each construct (Fig. 3B).

HeLa cells were transfected with these constructs, and the FRET efficiency was quantified using FACS 48 h post-transfection. The highest FRET efficiency was observed in the ER, followed by the mitochondria and nucleolus, suggesting elevated crowding in these compartments (Fig. 3C–E).

**Fig. 4:**
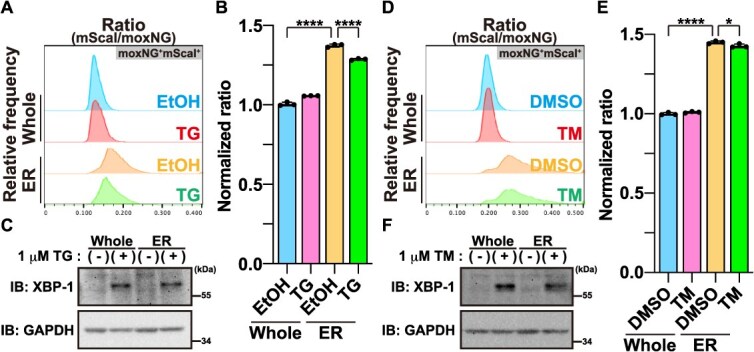
**ER stress is associated with reduced macromolecular crowding.** (A–C) HeLa cells were transfected with either moxCRONOS or moxCRONOS-ER. After 24 h, the cells were treated with 1 μM thapsigargin (TG) for 24 h, fixed, and analyzed by FACS. (A) Histograms showing the distribution of the mScarlet-I/moxNeonGreen (mScaI/moxNG) fluorescence intensity ratio within the moxNG^+^mScaI^+^ population. (B) Median values of the mScarlet-I/moxNeonGreen ratio calculated from the moxNG^+^mScaI^+^ population shown in (A). Ethanol (EtOH) was used as a negative control. XBP-1 splicing was confirmed by detecting spliced XBP-1 protein using western blotting analysis (C). (D–F) HeLa cells were transfected with either moxCRONOS or moxCRONOS-ER. After 48 h, cells were treated with 1 μM tunicamycin (TM) for 8 h, fixed, and analyzed by FACS. (D) Histograms showing the distribution of the mScarlet-I/moxNeonGreen (mScaI/moxNG) fluorescence intensity ratio within the moxNG^+^mScaI^+^ population. (E) Median values of the mScarlet-I/moxNeonGreen ratio calculated from the moxNG^+^mScaI^+^ population shown in (D). Dimethyl sulfoxide (DMSO) was used as a negative control. XBP-1 splicing was confirmed by detecting spliced XBP-1 protein using western blotting analysis (F).

To assess whether ER crowding is affected by ER stress, we treated cells expressing moxCRONOS-ER with thapsigargin (TG), an inhibitor of sarcoplasmic reticulum/ER Ca^2+^-ATPase (SERCA), which induces ER stress by disrupting calcium homeostasis. Following TG treatment, a modest but significant decrease in FRET efficiency was observed (Fig. 4A and B), indicating a reduction in molecular crowding within the ER. A similar decrease in FRET efficiency was also observed upon treatment with tunicamycin (TM), a glycosylation inhibitor that likewise induces ER stress (Fig. 4D and E). The induction of ER stress under these conditions was verified by detecting spliced XBP-1 (Fig. 4C and F). These results suggest that ER stress is accompanied by loosening of the macromolecular environment, potentially reflecting attenuated protein synthesis and remodeling of ER architecture.

### moxCRONOS functions as a sensor for protein aggregation

**Fig. 5:**
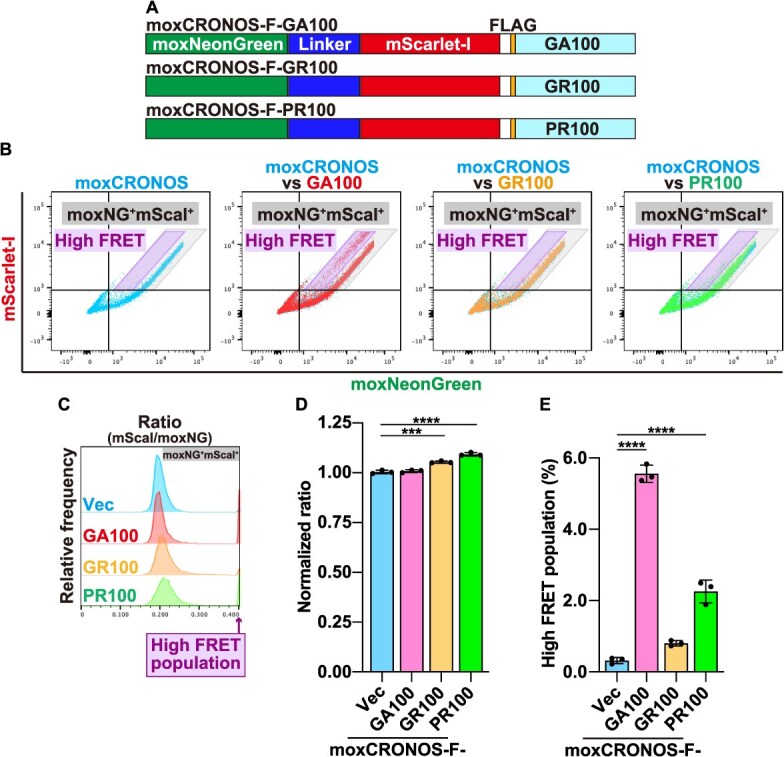
**moxCRONOS functions as a FRET-based sensor to detect protein aggregation.** (A) Schematic representation of moxCRONOS protein constructs in which FLAG (F)-tagged DPRs (GA_100_, GR_100_, PR_100_) derived from *C9ORF72* gene mutations are fused to the C-terminus of moxCRONOS. (B–D) HeLa cells were transfected with each moxCRONOS-DPR construct. After 48 h, cells were fixed and analyzed by FACS. (B) Dot plots show fluorescence intensities of moxNeonGreen (*x*-axis) and mScarlet-I (*y*-axis). (C) Histograms showing the distribution of the mScarlet-I/moxNeonGreen (mScaI/moxNG) fluorescence intensity ratio within the moxNG^+^mScaI^+^ population delineated by the gray parallelogram in (B). (D) Median values of the mScarlet-I/moxNeonGreen ratio calculated from the moxNG^+^mScaI^+^ population shown in (C). (E) Proportion of cells in the high-FRET region delineated by the purple parallelogram in (B).

In neurodegenerative disorders, abnormal phase separation can trigger aggregation of pathological proteins, leading to the formation of densely packed structures. We hypothesized that fusing moxCRONOS with aggregation-prone proteins would reveal dense environments through FRET. To test this, we created moxCRONOS constructs fused to polydipeptides associated with *C9ORF72* repeat expansions glycine–alanine (GA)_100_, glycine–arginine (GR) _100_, and proline–arginine (PR) _100_—each consisting of 100 dipeptide repeats (Fig. 5A) *(**14**)*.

**Fig. 6:**
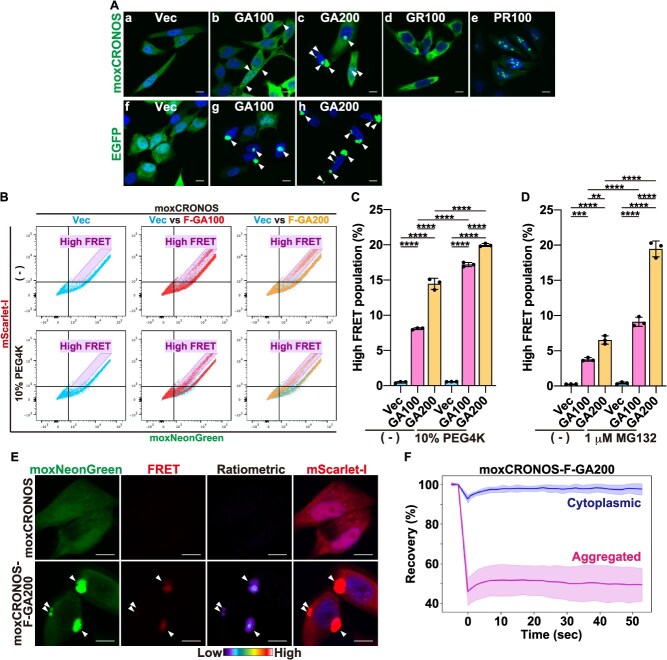
**moxCRONOS detects GA repeat length-dependent protein aggregation, which is further promoted by crowding and proteasome inhibition.** (A) HeLa cells were transfected with DPRs fused to either moxCRONOS or EGFP. After 48 h, cells were fixed and observed by confocal laser scanning microscopy. Arrowheads indicate aggregate formation. (B, C) HeLa cells were transfected with moxCRONOS, moxCRONOS-FLAG (F)-GA_100_, or moxCRONOS-F-GA_200_. After 24 h, cells were treated with 10% PEG4000 (PEG4K) for 24 h, fixed, and analyzed by FACS. (B) Dot plots show fluorescence intensities of moxNeonGreen (*x*-axis) and mScarlet-I (*y*-axis). (C) Proportion of cells in the high-FRET region delineated by the purple parallelogram in (B). (D) HeLa cells were transfected with moxCRONOS, moxCRONOS-F-GA_100_, or moxCRONOS-F-GA_200_. After 24 h, cells were treated with 1 μM MG132 for 24 h, fixed, and analyzed by FACS. The proportion of cells in the high-FRET region is quantified in the graph. (E) HeLa cells were transfected with moxCRONOS or moxCRONOS-F-GA_200_. After 24 h, cells were treated with 10% PEG4000 for 24 h, fixed, and observed by confocal laser scanning microscopy. Fluorescence from each protein and FRET signal were detected using the following excitation (Ex) and emission (Em) settings: moxNeonGreen: Ex. 488 nm, Em. 520–560 nm; FRET: Ex. 488 nm, Em. 590–712 nm; ratiometric: FRET/moxNeonGreen; mScarlet-I: Ex. 561 nm, Em. 568–712 nm. Arrowheads indicate aggregate formation. (F) HeLa cells were transfected with moxCRONOS-F-GA_200_ and subjected to FRAP analysis 24 h after transfection. Fluorescence recovery of cytoplasmic moxCRONOS-FLAG-GA_200_ (blue) and aggregated moxCRONOS-FLAG-GA_200_ (magenta) is shown. Fluorescence intensities were normalized to prebleach values, and recovery curves were generated. Solid lines represent the mean, and shaded areas indicate the SD. Ten cells were analyzed per condition.

In the main cell population, GA_100_-fused moxCRONOS showed no significant changes in FRET, GR_100_-fused moxCRONOS showed a slight increase, and PR_100_-fused moxCRONOS showed an intense FRET signal (Fig. 5B–D). This correlated with the nucleolar localization of PR_100_ (Fig. 6A–E), where the crowding level was relatively high (Fig. 3C–E).

Notably, a small subpopulation of cells expressing GA_100_-fused moxCRONOS exhibited a pronounced increase in FRET (Fig. 5B, C, and  E; high-FRET population), suggesting the formation of dense aggregation-like structures, as shown previously *(**9**)*. However, these aggregates were much smaller than those seen in EGFP-GA_100_-expressed cells (Fig. 6A, compare b and g). However, FACS analysis, with its higher sensitivity, was able to detect this small high-FRET subpopulation. The smaller and fewer moxCRONOS-GA_100_ aggregates may be due to the relatively large size and high solubility of moxCRONOS, which, when fused to GA_100_, could suppress the intrinsic aggregation propensity of GA repeats compared to smaller tags, such as EGFP. To enhance aggregate formation, the number of GA repeats was increased from 100 to 200. As expected, moxCRONOS-GA_200_ formed large aggregates, similar to EGFP-GA_200_ (Fig. 6A, compare c and h). FACS analysis confirmed that a greater proportion of cells exhibited high-FRET than moxCRONOS-GA_100_ (Fig. 6B and C).

Importantly, the high-FRET population increased further upon treatment with PEG4000 (Fig. 6B and C), which promoted molecular crowding, and MG132 (Fig. 6D), a proteasome inhibitor that stabilizes GA aggregates *(**15**)*. Confocal microscopy confirmed that high-FRET cells had aggregate-like structures (Fig. 6E). Fluorescence recovery after photobleaching (FRAP) analysis revealed that these structures were aggregates, exhibiting minimal recovery (Fig. 6F). These findings indicate that moxCRONOS is a useful biosensor not only for sensing macromolecular crowding but also for detecting aggregation-prone protein states.

## Discussion

In this study, we developed and validated moxCRONOS, a monomeric, oxidation-resistant variant of the CRONOS crowding sensor. By substituting a cysteine residue (C149) with serine, moxCRONOS avoids oxidative modifications, preserving sensor performance in oxidative environments, such as the ER and secretory pathway. The ER represents an oxidizing environment *(**16**)*, and mitochondria maintain distinct redox states from the cytosol *(**17**,*  *18**)*. The redox-insensitive design of moxCRONOS enables direct comparison of macromolecular crowding across subcellular compartments, independent of redox conditions. Spectroscopy confirmed that moxCRONOS retained the FRET-based sensing capability of CRONOS, as demonstrated by the PEG4000-induced spectral shift and nearly identical emission profiles. moxCRONOS retained stable performance under the oxidative conditions, demonstrating that removal of this reactive thiol effectively prevents redox interference. Thus, moxCRONOS preserves sensitivity to macromolecular crowding while avoiding oxidative artifacts, making it a reliable tool for probing crowding in oxidizing compartments such as the ER or during cellular stress.

Targeting moxCRONOS to organelles allowed the mapping of relative crowding, with the ER showing the highest FRET efficiency, followed by mitochondria and nucleolus. These findings suggest that these compartments may have a particularly high macromolecular density, likely due to their respective functions: protein folding and secretion in the ER *(**19**)*, ATP production and metabolic activity in mitochondria *(**20**)*, and ribosomal biogenesis in the nucleolus *(**21**)*. Thus, moxCRONOS provides a reliable tool for assessing physiologically relevant crowding.

Importantly, we observed that ER stress inducers significantly decreased FRET efficiency of ER-localized moxCRONOS, suggesting reduced molecular crowding. This implies that ER stress may involve biochemical changes, as well as biophysical remodeling of the ER lumen. One possibility is that the unfolded protein response (UPR), which is activated during ER stress, expands ER luminal volume as a compensatory mechanism to alleviate crowding and facilitate protein folding *(**22**)*. Such an expansion could buffer the accumulation of unfolded proteins by decreasing the local macromolecular density *(**23**)*. Moreover, the UPR reduces global protein synthesis, which may also contribute to reduced crowding. Together, these mechanisms likely cooperate to relieve ER burden, and moxCRONOS has the potential to detect such dynamic biophysical adaptations under stress conditions, offering novel insights into proteostasis regulation.

moxCRONOS also proved to be a valuable reporter of pathological protein aggregation, particularly in *C9ORF72*-associated DPRs. The fusion of moxCRONOS to the GA_100_, GR_100_, and PR_100_ dipeptides revealed differential FRET signals, with PR_100_ exhibiting the highest FRET, consistent with its nucleolar localization and elevated crowding in this compartment. This may reflect multivalent interactions mediated by arginine residues in PR repeats, promoting assembly and phase separation *(**24**)*. Although most GA_100_-expressing cells showed no FRET changes, a minor subpopulation displayed markedly increased FRET, likely representing subpopulations harboring protein aggregates. This highlights the ability of FACS to detect heterogeneous events. To enhance the aggregation detection, we generated moxCRONOS-GA_200_, which successfully formed large aggregates and yielded a greater proportion of high-FRET cells using FACS. Aggregation-promoting treatments, such as PEG4000 and MG132, further increased FRET efficiency. These results confirm that moxCRONOS sensitively detects both physiological crowding and aberrant aggregation.

Although moxCRONOS successfully detected increased FRET signals associated with aggregation, one limitation of this study is that we could not clearly determine whether the FRET increase arose from intramolecular structural changes or intermolecular proximity. Because aggregation brings many sensor molecules into close proximity, intermolecular FRET can occur, potentially complicating the interpretation of crowding versus aggregation. To address this challenge, future efforts should focus on developing improved sensor designs that restrict FRET to within a single molecule—for example, by optimizing the distance and relative orientation between the two fluorescent proteins. Such orientation-controlled sensors would help distinguish genuine crowding-induced conformational changes from FRET signals arising from aggregation-driven clustering of the sensor.

In this study, we also considered the possibility that fusion with moxCRONOS may affect the solubility and aggregation behavior of the target proteins. Indeed, moxCRONOS-GA_100_ tended to form smaller and fewer aggregates compared with EGFP-GA_100_, which may reflect the bulky size of the sensor and the relatively high solubility of CRONOS. Nevertheless, when the GA repeat length was increased to 200 or when cells were treated with PEG4000 or MG132, large aggregates and distinct high-FRET cell populations were readily detected. These results suggest that moxCRONOS functions not as an oversensitive reporter but rather as a ‘conservative sensor’ that may underestimate, rather than exaggerate, aggregation behavior. Such a property could be advantageous by avoiding false positives and selectively capturing pathologically meaningful aggregation events. In the future, further improvements such as the use of smaller FRET pairs or designs restricted to intramolecular FRET may help to minimize potential perturbations to the target proteins.

The collective findings establish moxCRONOS as a versatile biosensor for quantitative, compartment-specific analysis of macromolecular crowding and protein aggregation in living cells. Its monomeric and oxidation-resistant design makes it particularly well-suited for use in oxidative environments where traditional sensors often fail. moxCRONOS holds significant promise for future studies of cellular stress responses, phase separation dynamics, neurodegenerative disease models, and therapeutic screening efforts targeting protein homeostasis.
